# Primary hyperparathyroidism diagnosed after surgical ablation of a costal mass mistaken for giant-cell bone tumor: a case report

**DOI:** 10.1186/1752-1947-5-596

**Published:** 2011-12-28

**Authors:** Lara Vera, Mara Dolcino, Marco Mora, Silvia Oddo, Marina Gualco, Francesco Minuto, Massimo Giusti

**Affiliations:** 1 Department of Internal Medicine, University of Genoa, Viale Benedetto XV, 6-16132, Genoa, Italy; 2 Department of Pathology, National Institute for Cancer Research, Genoa, Italy

## Abstract

**Introduction:**

Primary hyperparathyroidism is a common endocrine disorder characterized by elevated parathyroid hormone levels, which cause continuous osteoclastic bone resorption. Giant cell tumor of bone is an expansile osteolytic tumor that contains numerous osteoclast-like giant cells. There are many similarities in the radiological and histological features of giant cell tumor of bone and brown tumor. This is a rare benign focal osteolytic process most commonly caused by hyperparathyroidism.

**Case presentation:**

We report the unusual case of a 40-year-old Caucasian woman in which primary hyperparathyroidism was diagnosed after surgical ablation of a costal mass. The mass was suspected of being neoplastic and histopathology was compatible with a giant cell tumor of bone. On the basis of the biochemical results (including serum calcium, phosphorous and intact parathyroid hormone levels) primary hyperparathyroidism was suspected and a brown tumor secondary to refractory hyperparathyroidism was diagnosed.

**Conclusions:**

Since giant cell tumor is a bone neoplasm that has major implications for the patient, the standard laboratory tests in patients with bone lesions are important for a correct diagnosis.

## Introduction

Primitive hyperparathyroidism (PHPT) is the third most common endocrine disorder after diabetes mellitus and thyroid dysfunction [1]. The estimated incidence of cases of PHPT is 0.2% to 0.3% [2]. The diagnosis of PHPT has classically been based on the demonstration of high plasma calcium and low plasma phosphorus concentrations. In recent years, however, it has been recognized that patients with PHPT may present with plasma calcium concentrations within the normal range [3]. About 75% to 80% of cases of PHPT are diagnosed when a routine assay shows hypercalcemia in patients who are asymptomatic or during evaluation for osteoporosis. Surgical ablation is the treatment of choice for PHPT. Persistent hyperparathyroidism leads to altered osseous metabolism involving bone resorption and tissue changes that are collectively known as osteitis fibrosa cystica (OFC) [3]. Today, < 5% of patients display evidence of OFC [4]. Osteitis fibrosa cystica is characterized by the presence of subperiosteal resorption in the digits, skull and long bones, diffuse osteopenia, and brown tumor [5].

Brown tumor is an extremely rare osseous lesion that constitutes a focal manifestation of OFC induced by hyperparathyroidism, independently of its cause. The reported prevalence of brown tumors is 0.1%, and they have been reported to occur in 4.5% of patients with PHPT and in 1.5% to 1.7% of those with secondary disease [6,7]. The disease can manifest itself at any age, but is more common among people older than 50 years, and is three times more common in women than in men [8]. Brown tumor may be the first clinical sign of hyperparathyroidism. Histologically, brown tumors are made up of mononuclear stromal cells mixed with multinucleated giant cells, among which recent hemorrhagic infiltrates and hemosiderin deposits (hence the brown color) are often found [9,10]. Brown tumors may appear in any of the bones [11]. However, when the same type of lesion is found in patients without PHPT, the differential diagnosis becomes more complex.

Many of the radiological and histological features of brown tumor, a benign osteolytic process, are similar to those of giant cell tumor (GCT) of bone. Giant cell tumor of bone is an uncommon primary bone tumor that accounts for around 5% of all primary bone tumors [12]. Giant cell tumor is a locally aggressive tumor characterized by a high number of multinuclear giant cells that exhibit the features of mature osteoclasts [13]. Clinically, most patients with GCT are asymptomatic or present with bone pain due to enlargement of the tumor. Malignant transformation of GCT is a rare event, occurring in less than 1% of all cases [14]. The treatment of primary GCT is essentially surgical [12]. The differential diagnosis is based on biochemical analysis.

Here, we report the case of a patient with an incidentally discovered costal mass, the diagnostic investigation of which led to an unexpected diagnosis.

## Case presentation

We describe the case of a 40-year-old Caucasian woman who had first been examined at the Department of Internal Medicine of our University Hospital three years ago for a Reynaud-like disorder. Her medical history was unremarkable except for an oligomenorrhea since she was 26 years of age. A physical examination revealed a fair condition. A chest X-ray showed a peripheral lesion, approximately 8 cm in diameter, of the third right rib with cortical expansion; this was confirmed by a contrast tomography scan. The lesion was associated with an area of bone rarefaction of the lamina of L2. The bone mass was suspected of being a neoplastic lesion. Subsequently,^m99^Tc bone scintigraphy revealed a hyper-metabolic focus in the rib and in the medial condylus of the right femur, while an magnetic resonance imaging (MRI) scan showed an aneurysmal cystic bone lesion. Costal biopsy showed numerous osteoclast-like giant plurinuclear cells, without necrosis, mitoses or histological signs of malignancy; a picture compatible with GCT of bone. Six months after diagnosis, the mass was surgically removed and local curettage was performed, with excision of the right major pectoralis and part of the II, III and IV right ribs. Histology showed a neoplasm with a solid structure made up of bland mononuclear stromal cells and an osteoclast-type giant cell-rich component, a picture compatible with GCT.

**Figure 1:**
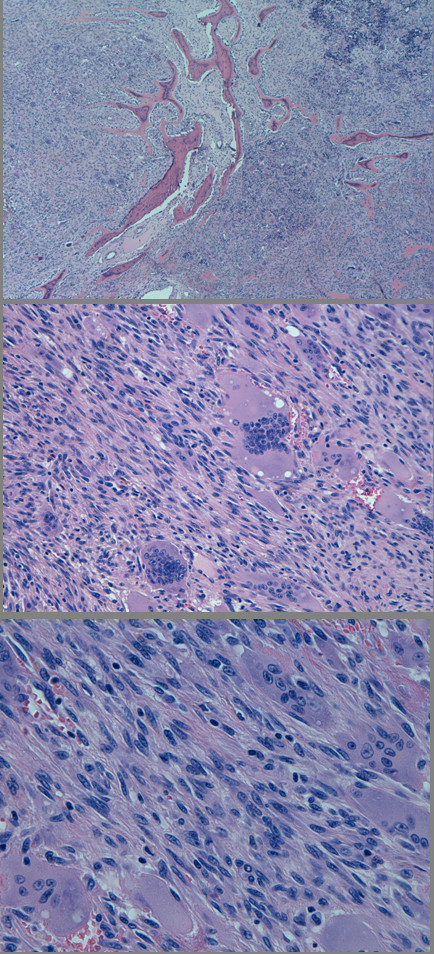
**Histopathological analysis of the surgical specimens**. Top: a solid mass with diffuse infiltration and destruction of bone tissue (hematoxilin and eosin (H&E), original magnification 4×). Center: the solid mass is composed of numerous multinucleated giant cells in a background of middle-sized cells (H&E, original magnification 20×). Bottom: at higher magnification, the background spindle-cell component is seen to be composed of mononuclear spindle-shaped or oval-shaped cells, with eosinophilic cytoplasm and oval nuclei without atypia (H&E, original magnification 40×).

On gross examination, the surgical specimen consisted of a soft, well circumscribed endothoracic mass of 6 × 4.5 × 2.2 cm, with a tan and fleshy cut surface, which conglobated two ribs (11 cm and 5.5 cm in length). On microscopic examination, the tumor was seen to be composed of a large number of single multinucleated giant cells diffusely distributed on a background of spindle-shaped, oval or polygonal mononuclear cells; these latter cells were characterized by abundant eosinophilic cytoplasm and oval nuclei without atypia. No aggregates of giant cells were observed. Necrosis and areas of hemorrhage were not observed. The mononuclear component displayed a low mitotic index (6/10 high-power field [HPF]) (Figure 1).

**Figure 2:**
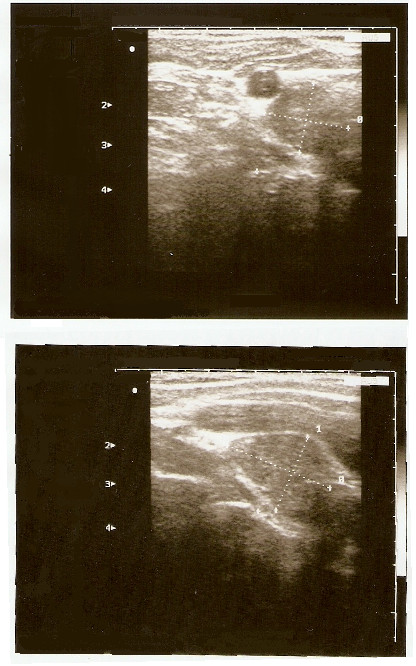
**Neck ultrasonography images obtained about 21 months previously, demonstrating a solid cervical mass of 14 × 16 × 27 mm, with intra-lesional vascularization, under the right thyroid lobe**. After fine-needle aspiration biopsy (FNAB), parathyroid hormone (PTH) evaluation on fine-needle washing was 550 pg/mL; this value was higher than our institutional standard cut-off value of 132 pg/mL [30].

**Figure 3:**
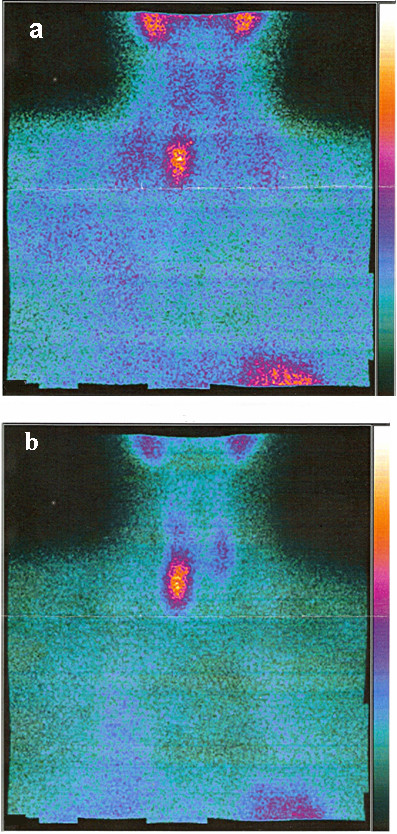
**Cervical scintigraphy obtained at the baseline (A) and two hours after (B) 185 mBq of^99 m^T-sestamibi injection about 22 months previously; accumulation of the isotope reflects the hyperfunctioning parathyroid adenoma**.

One month after surgery, during the first medical and laboratory follow-up examination, an increase in serum calcium (3.6 mmol/L) and alkaline phosphatase (ALP: 501 U/L) levels was found. Our patient's parathyroid function was found to be elevated (parathyroid hormone [PTH]: 712 ng/L). Our patient was referred for endocrine evaluation two months after surgery. Review of her medical records revealed hypercalcemia, which had been underestimated at the first examination. Her family history was negative for parathyroid and other endocrine diseases. PHPT was suspected. No cervical masses were palpable. Neck ultrasonography identified a round lesion located behind and below the right thyroid lobe and compatible with an enlarged parathyroid gland, whereas the thyroid was normal (Figure 2). A^99 m^Tc-sestamibi scan revealed a hyper-functioning area in the upper right portion of the thyroid bed, which was suspected of being an underlying parathyroid adenoma (Figure 3). Cytological examination of a fine-needle aspiration biopsy of the nodular lesion was compatible with a parathyroid adenoma. Sequencing of the multiple endocrine neoplasia (MEN)-1 gene proved normal. All biochemical data observed before parathyroidectomy are reported in Table 1 and in the legend to Figure 2. A mini-invasive parathyroidectomy was scheduled. Histology confirmed the diagnosis of adenoma of the right inferior parathyroid gland. Post-operative laboratory tests showed normal levels of serum calcium (2.3 mmol/L) and PTH (80 ng/L). After a review of bone slices, the final diagnosis of the bone disease was a brown tumor secondary to probably long-standing underestimated PHPT. At present, one year later, our patient is free from symptoms and dual-emission X-ray absorptiometry (DXA) has shown that her bone density has improved (Table 1).

**Table 1:** Some laboratory data and T scores data from dual-emission X-ray absorptiometry analysis performed before and after parathyroidectomy

	Admission	Before bone tumor surgery	After bone tumor surgery	Before parathyroidectomy	After parathyroidectomy	1 year post-parathyroidectomy
Ca (mmol/L), normal value 2.1 to 2.7	3.7	3.6	3.6	3.2	2.3	2.3
P (mmol/L), normal value 0.81 to 1.45	0.55	0.48	0.68	0.48	-	0.81
ALP (U/L), normal value 98 to 280	347	501	423	224	-	192
PTH (ng/L), normal value 17 to 73	-	-	712	873	88	80
Creatinine (μmol/L), normal value 45 to 120	70	-	-	70	-	-
25[OH]D (μg/L), normal value 6 to 46	-	-	-	13	8.2	9.9
1,25(OH)_2_D (pmol/L), normal value 48 to 110	-	-	-	212.3	70.7	101.7
Osteocalcin (μg/L), normal value 6.5 to 42.3	-	-	-	60.3	-	14.1
T-score, L2 to L4	-	-	-	-2.48	-1.63	-1.25
T-score, femoral	-	-	-	-1.91	-1.29	-0.99

1,25(OH)_2_D, 1,25-dihydroxyvitamin D_3_; 25[OH]D, 25-hydroxyvitamin D; ALP, alkaline phosphatase; PTH, parathyroid hormone.

## Discussion

Primitive hyperparathyroidism involves a heterogeneous group of patients with various symptoms and pathological conditions.

Brown tumor is an unusual non-neoplastic lesion resulting from abnormal bone metabolism in hyperparathyroidism and represents a reparative cellular process [15] that arises from foci of OFC, the end stage of a bone-remodeling condition. Brown tumor is mainly due to secondary hyperparathyroidism in patients with renal insufficiency, but it has also been described as an extremely rare manifestation of PHPT [11], usually resulting from PTH overproduction by adenomas [16] or carcinomas [17] of the parathyroid glands. This particular manifestation of PHPT appears in advanced stages of the disease. In contrast with these previous reports, in our patient's case there was no evidence of advanced end-stage disease. Moreover, our patient was only 40 years of age and presented with only slight osteopenia without other symptoms or signs of malnutrition. However, she showed typical laboratory findings of PHPT.

Increased PTH levels induce the proliferation and differentiation of pluripotent bone-marrow cells into osteoblasts and induce the migration and differentiation of monocytes into osteoclasts; the increased number of the latter in the bone tissue [3,6] causes bone resorption to predominate over the formation of new bone tissue. Brown tumor therefore involves a combination of osteoblastic and osteoclastic activity.

Brown tumors were first reported about two decades ago in 1.5% to 1.7% of patients with secondary hyperparathyroidism and in 3% of those with PHPT [6,18].

Our patient's case of PHPT is probably sporadic, as the majority of parathyroid tumors are sporadic. Only 5% are associated with hereditary endocrine tumor syndrome, which includes such diseases as MEN-1, MEN-2, familial benign hypocalciuric hypercalcemia and familial isolated PHPT.

These lesions may appear in any part of the skeleton and one or more bones may be involved. Lesions are generally located in areas of intense bone resorption [19].

Localized brown tumors are difficult to diagnose because they present clinically and radiologically as expansile multi-locular masses. The upper end of the tibia is the most common site of these lesions; unusual sites include the pelvic bone, mandible, ribs, femur and vertebral body.

In our patient, serum 25-hydroxyvitamin D (25[OH]D) was low, and it is well known that, in asymptomatic PHPT, low 25[OH]D levels are associated with increased disease activity [20]. The tumors may cause pain and/or fractures. Symptoms depend on the size and location of the lesions. In other cases, lesions have been asymptomatic, the diagnosis following incidental radiological detection.

Histopathological analysis of the osseous lesion is needed in order to confirm the diagnosis of a brown tumor. As brown tumors exhibit no pathognomonic histological changes, it may be very difficult to distinguish a brown tumor from other giant-cell tumors, even on histological examination. In our patient, the histological findings suggested a giant-cell expanding bone lesion. It is important to point out that brown tumors are non-neoplastic lesions that are very similar to GCT; in the context of PHPT, they are considered reparative granulomas and do not have the malignant or neoplastic potential of true giant-cell lesions.

On X-ray imaging, brown tumors appear as lytic lesions with tortuous contours. Concurrent changes, such as osteopenia, a 'salt-and-pepper' bone appearance and subperiostal bone resorption, suggest OFC. Tomography shows an osseous mass and areas that appear cystic [21]. MRI may be better able to determine the presence of cysts. On^99 m^Tc-sestamibi, isolated hypermetabolic lesions and the simultaneous hypercaptation of bone lesions and parathyroid adenomas have been described [22].

The main differential diagnosis is with GCT [6,18], which is a highly vascular lesion. The radiological appearance and, even more so, the histology of GCT may closely mimic brown tumor, as in our patient. As it is very difficult to distinguish brown tumors from other giant-cell lesions histologically or radiologically, a definitive diagnosis is only possible on comparing the clinical manifestations and radiological and laboratory test results that differentiate the lesions. Thus, the clinical diagnosis is made in relation to PHPT [7,10].

The first step in the management of brown tumor is to treat PHPT, which may be performed by means of parathyroidectomy. The effect of parathyroidectomy on brown tumors depends on their composition [23]. After treatment of the underlying metabolic disorder, the regression or complete remission of brown tumors has been well documented [24] in primary and secondary hyperparathyroidism. Patient age appears to be an important factor in predicting the time required for brown tumors to regress. Several authors consider parathyroidectomy the only correct therapy [24]. However, several cases have been reported of brown tumors that grew after parathyroidectomy or normalization of PTH levels [25]. In these cases, resection of the brown tumor should be the treatment of choice. Some authors [26] believe that bone lesions reappear spontaneously following removal of the diseased parathyroid gland; others [27] recommend initial treatment with systemic corticosteroids to reduce tumor size, followed by surgical removal of the residual lesion. Surgical resection and decompression of brown tumors should be considered if the patient is symptomatic [28]. In cases in which lesions persist for more than six months, or there is disruption of the function of the affected organ, or growth despite adequate metabolic control, some authors [25] have recommended tumor curettage and associated enucleation. Finally, vitamin D improves serum PTH levels, and medical treatment based on high doses of vitamin D is effective in many cases of brown tumor [29].

## Conclusions

In summary, we report the case of a 40-year-old woman with the association of brown tumor and a single parathyroid adenoma, in whom a bone neoplasm was first suspected. The correct diagnosis was reached only later, despite the many clinical manifestations. Radiograph studies showed a combination of typical changes. This case highlights the many similarities between GCT and brown tumor.

## Consent

Written informed consent was obtained from the patient for publication of this case report and any accompanying images. A copy of the written consent is available for review by the Editor-in-Chief of this journal.

## Competing interests

The authors declare that they have no competing interests.

## Authors' contributions

LV analyzed and interpreted the data from our patient regarding endocrinological disease; MD and SO were contributors to writing the manuscript; MM and MGU performed the histological examination of bone samples; MGI was a major contributor to writing the manuscript. All authors read and approved the final manuscript.
